# The clonal expression genes associated with poor prognosis of liver cancer

**DOI:** 10.3389/fgene.2022.808273

**Published:** 2022-08-25

**Authors:** Wanfeng Zhang, Fang Huang, Xia Tang, Longke Ran

**Affiliations:** ^1^ Department of Bioinformatics, Basic Medical College, Chongqing Medical University, Chongqing, China; ^2^ Fudan University, Shanghai, China

**Keywords:** liver cancer, intratumor heterogeneity (ITH), clonal, prognosis, evolution

## Abstract

The extensive spatial genomic intratumor heterogeneity (ITH) in liver cancer hindered treatment development and limited biomarker design. Early events that drive tumor malignant transformation in tumor founder cells are clonally present in all tumor cell populations, which provide stable biomarkers for the localization of tumor cells and patients’ prognosis. In the present study, we identified the recurrently clonal somatic mutations and copy number alterations (CNAs) (893 clonal somatic mutations and 6,617 clonal CNAs) in 353 liver cancer patients from The Cancer Genome Atlas (TCGA) and evaluated their prognosis potential. We showed that prognosis-related clonal alterations might play essential roles in tumor evolution. We identified 32 prognosis related clonal alterations differentially expressed between paired normal and tumor samples, that their expression was cross-validated by three independent cohorts (50 paired samples in TCGA, 149 paired samples in GSE76297, and 9 paired samples in SUB6779164). These clonal expression alterations were also significantly correlated with clinical phenotypes. Using stepwise regression, we identified five (*UCK2*, *EFNA4*, *KPAN2*, *UBE2T*, and *KIF14*) and six (*MCM10*, *UCK2*, *IQGAP3*, *EFNA4*, *UBE2T*, and *KPNA2*) clonal expression alterations for recurrence and survival model construction, respectively. Furthermore, in 10 random repetitions, we showed strong applicability of the multivariate Cox regression models constructed based on the clonal expression genes, which significantly predicted the outcomes of the patients in all the training and validation sets. Taken together, our work may provide a new avenue to overcome spatial ITH and refine biomarker design across cancer types.

## Introduction

Liver cancer, especially hepatocellular carcinoma (HCC), is the leading cause of cancer-related death worldwide (Bray et al., 2018), and thus an early prognostic evaluation is an important measure to improve clinical management. Although multiple attempts have been made to design an effective prognostic biomarker for liver cancer patients (Villanueva et al., 2011; Cai et al., 2017), seldom of them have been adopted in clinical practice due to poor reproducibility or worse efficiency than the clinic-pathological risk factors (Marques et al., 2020).

All cells within the tumor are unique and continue to acquire new alterations in the evolution process (Lynch, 2010; Martincorena and Campbell, 2015), resulting in considerable genomic intratumor heterogeneity (ITH). The ITH may be the leading cause of inefficient biomarker design. Previous studies revealed that HCC tumors showed an extensively spatial ITH, displaying a clear isolation-by-distance pattern where spatially greater sectors are genetically more different (Zhai et al., 2017). The particular isolated growth pattern results in significant spatial ITH of the genome, epigenome, copy number alterations (CNAs), and transcriptome, which will bring significant sampling bias. Based on multi-region sequencing, studies revealed the molecular biomarkers might be confounded by sampling bias arising from ITH in transcription level (Gerlinger et al., 2012; Gulati et al., 2014, 2015; Gyanchandani et al., 2016; Lee et al., 2018; Biswas et al., 2019). Therefore, addressing the impact of ITH on biomarker design is a fundamental challenge for precision medicine (Barranco et al., 1994; Blackhall et al., 2004; Bachtiary et al., 2006; Boutros, 2015).

In tumor evolution, early genomic alterations in tumor founder cells are stably inherited in all tumor cell populations, which present as clonal and overcome the spatial ITH. These clonal alterations provide not only promising therapeutic targets but also stable biomarkers. Evidence showed that some tumors were born-to-be-bad, where the malignant potential is specified early in colorectal cancer (Sottoriva et al., 2015; Ryser et al., 2018). In HCC, early genomic divergence also demonstrated early malignant characteristics (Zhai et al., 2017; Ding et al., 2019). This evidence revealed that early clonal alterations that are localized in all tumor cell populations have potential prognostic value.

In the present study, we aimed to identify a set of prognosis-related clonal biomarkers acquired at early tumor progression, which kept prognosis potential both at genomic and transcriptome levels and may overcome the spatial ITH. Our research may bring a new avenue for the design of tumor biomarkers.

## Materials and methods

### Data collection

The somatic mutation data (pipelines, “mutect2”; reference genome, hg38) of liver cancer was downloaded by R (version, 4.0.5) package “TCGAbiolink” (Colaprico et al., 2016). The GISTIC score of copy number alterations (CNAs), normalized RNA expression, and clinical phenotype data were downloaded at The Cancer Genome Atlas (TCGA, xenabrowser.net). GSE76297 (149 paired normal and tumor HCC samples) and GSE10141 (80 HCC samples with follow-up data) were downloaded from the GEO database (https://www.ncbi.nlm.nih.gov/geo/).

### RNA sequencing

RNA sequencing was performed on nine liver tumor and paired non-tumor samples (Zhang et al., 2020). A detailed description of the pipeline for bioinformatics analysis has been described previously (Ding et al., 2015; Zhang et al., 2020). RNA sequencing data can be obtained from the Sequence Read Archive (SRA) (https://submit.ncbi.nlm.nih.gov/) (SUB6779164).

### Identification of clonal altered genes

Using R (version, 4.0.5) package “DoAbsolute” (Carter et al., 2012; Wang, 2021), we inferred the tumor’s purity, ploidy, and the cancer cell fraction (CCF), and clonality of each genomic alteration (including somatic mutations and CNAs). The parameters were used as follows, the “primary.disease” was “Hepatocellular Carcinoma,” “min.mut.af” was 0.05, “max.as.seg.count” was 5,000, “copy.num.type” was “total,” “platform” was “Illumina_WES,” and the remaining parameters were used as recommended of the package. For each alteration, it can be classified as a clone or subclone according to its CCF. The clonal alterations are present in all tumor cells and therefore have a higher CCF, whereas the subclonal alterations are only present in some tumor cells and therefore have a lower CCF. To obtain high-quality clonal alterations, we set a strict threshold that 1) the mutation was inferred as clonal mutation; 2) the lower confidence interval of CCF was at least 0.5; and 3) the events occurred at least in five patients.

### Assessment of the prognostic potential of each clonal alteration

According to the clonality of an alteration, the patients were divided into two groups (with or without this clonal alteration). We then evaluated the prognostic potential (overall and recurrence-free survival) of each clonal alteration using univariate Cox regression analysis in the “survival” package in R (version, 4.0.5) (Therneau and Grambsch, 2000). All the clonal alterations with a *p*-value < 0.05 were considered with prognostic potential (recurrence and survival). Gene Ontology analysis was performed to analyze the critical biological processes of these clonal alterations using the ‘clusterProfile’ package (Yu et al., 2012). Ten canonical oncogenic signaling pathways were obtained from the previous study (Sanchez-Vega et al., 2018).

### Identification of clonal expression genes

Paired T test was used to identify the differentially expressed genes (DEGs) between paired normal and tumor samples. All the *p*-value were adjusted by the false discovery rate (FDR). The DEGs that were cross-validated in the three cohorts (50 paired samples in TCGA, 149 paired samples in GSE76297, and 9 paired samples in our cohort) were considered significant DEGs. The prognosis related clonal alterations that also were DEGs in RNA level were selected for further analysis. Using the expression data, we evaluated the prognostic potential (overall and recurrence-free survival) of each DEG using univariate Cox regression analysis in the ‘survival’ package (Therneau and Grambsch, 2000). The correlation between GISTIC score and RNA expression was calculated by Pearson’s correlation analysis. The DEGs that both showed prognosis potential in clonal alterations and RNA expression were selected as candidate genes. The log2 (fold change) and log10 (hazard ratio) of each DEG was scaled by the formula: X=(X-Xmin)/(Xmax-Xmin), where X was the value of the log2 (foldchange) or log10 (hazard ratio), Xmax and Xmin were the max and min value of X, respectively. Furthermore, using a student t-test, we evaluated the difference of each candidate gene (RNA level) in clinical phenotypes, e.g., age, gender, Child-Pugh grade, liver fibrosis, histologic stage, American Joint Committee on Cancer (AJCC) stage, family cancer history, recurrence, alcohol history, HBV and HCV infection, and nonalcoholic fatty liver disease (NAFLD).

### Protein-protein interaction network construction and functional enrichment analysis

The genes showed directly interact with clonal expression genes in a high-quality interaction database (HINT) (Das and Yu, 2012) were selected for PPI network construction. Using the “clusterProfile” package (Yu et al., 2012), functional enrichment analysis was performed for these genes (KEGG pathways and Gene Ontology analysis).

### Multivariate cox regression model construction

All the clonal expression genes were assessed by a proportional hazard hypothesis test using the “survival” package (Therneau and Grambsch, 2000). Time-dependent variables were processed by constructing a function of time. Then, the prognosis-related clonal expression genes were used to construct a multivariate Cox regression model, and stepwise regression was used to select the clonal expression genes in the most minimalistic model. The best cutoff value for dividing patients into high and low-risk groups was calculated using the “ggrisk” package (Zhang and Jin, 2020). Furthermore, we randomly divided 70% of the patients into the training set to build the Cox regression model (using the selected clonal expression genes) and 30% as the validation set to evaluate the prognostic potential of the model. This process was repeated ten times in survival and recurrence models, respectively. The receiver operating characteristic (ROC) curve was used to evaluate the accuracy of patients predicted to be high-risk and has a poor prognosis. The Kaplan-Meier survival (KM) curve was used to analyze the difference between high- and low-risk groups.

### Statistical analysis

All the statistical analyses and plotting in this work were performed using R (version 4.0.5). *p* < 0.05 were considered statistically significant, and all the multiple testing correction used was performed using a false discovery rate (FDR).

## Results

### The clonality of each altered gene

**FIGURE 1:**
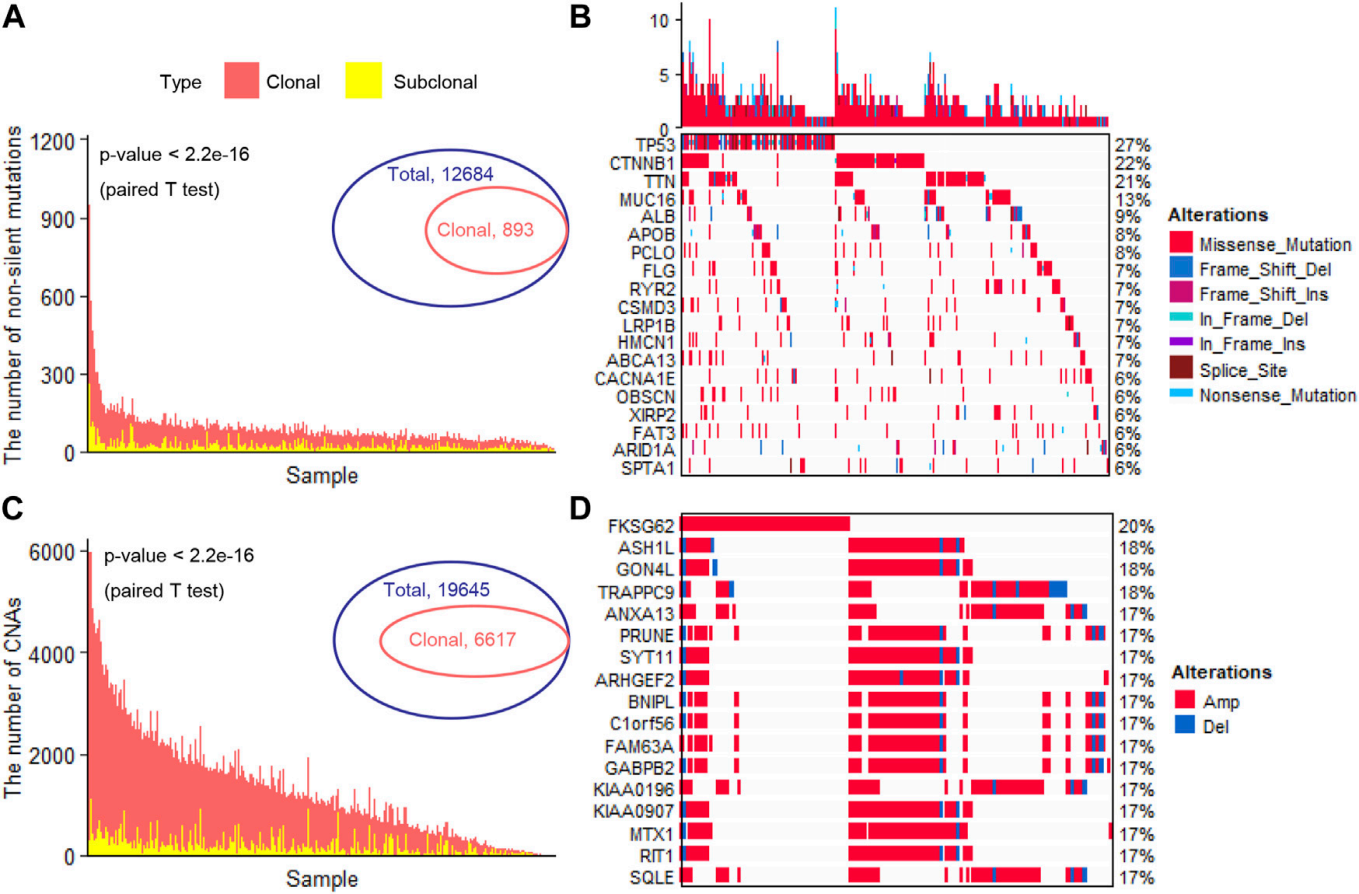
Identification of clonal altered genes **(A)**. The number of clonal and subclonal non-silent somatic mutations in each patient **(B)**. The landscape of the frequently clonal altered somatic mutations **(C)**. The number of clonal and subclonal CNAs in each patient **(D)**. The landscape of the frequently clonal altered CNAs. CNAs, copy number alterations; Amp, Amplification; Del, Deletion.

We totally observed 12,684 specific non-silent somatic mutated genes in 353 liver cancer patients (Figure 1A). In a single patient, the number of non-silent somatic mutations ranged from 11 to 1250 (median, 74) (Figure 1A). After adjusting the effects of the tumor’s purity and ploidy, we inferred the clonality of each mutation. Under a strict threshold (see Methods), we identified 893 specific clonal non-silent somatic mutated genes (ranged from 5 to 234 per patient; median, 15) (Figure 1A), including the known frequent liver cancer driver genes *TP53* (Chasov et al., 2020) (occurred in 96 patients, 27.3%), *CTNNB1* (76 patients, 21.6%), *TTN* (72 patients, 20.1%), *MUC16* (46 patients, 13.1%) and *APOB* (28 patients, 8.0%) (Tate et al., 2019) (Figure 1B). In each patient, we identified a considerable number of subclonal non-silent mutations (ranging from 0 to 260; median, 15) (Figure 1A), suggesting a continuous evolution after malignant transformation. Under the same threshold, we identified 6,617 specific clonal altered genes with significant CNAs (ranged from 2 to 2,346 per patient; median, 304) (Figure 1C), and the frequently altered genes were *FKSG62* (71 patients, 20.1%), *ASH1L* (64 patients, 18.1%), *GON4L* (65 patients, 18.4%), *TRAPPC9* (64 patients, 18.1%), *ANXA13* (61 patients, 17.3%) and *SYT11* (6 patients, 17.3%) (Figure 1D). We observed a large number of clonal and a small number of subclonal CNAs in most patients (Figure 1C), consistent with previous studies that most of the CNAs occurred early during tumor initiation (Navin et al., 2011; Wang et al., 2014; Duan et al., 2018).

### The prognostic potential of the clonally altered genes

**FIGURE 2:**
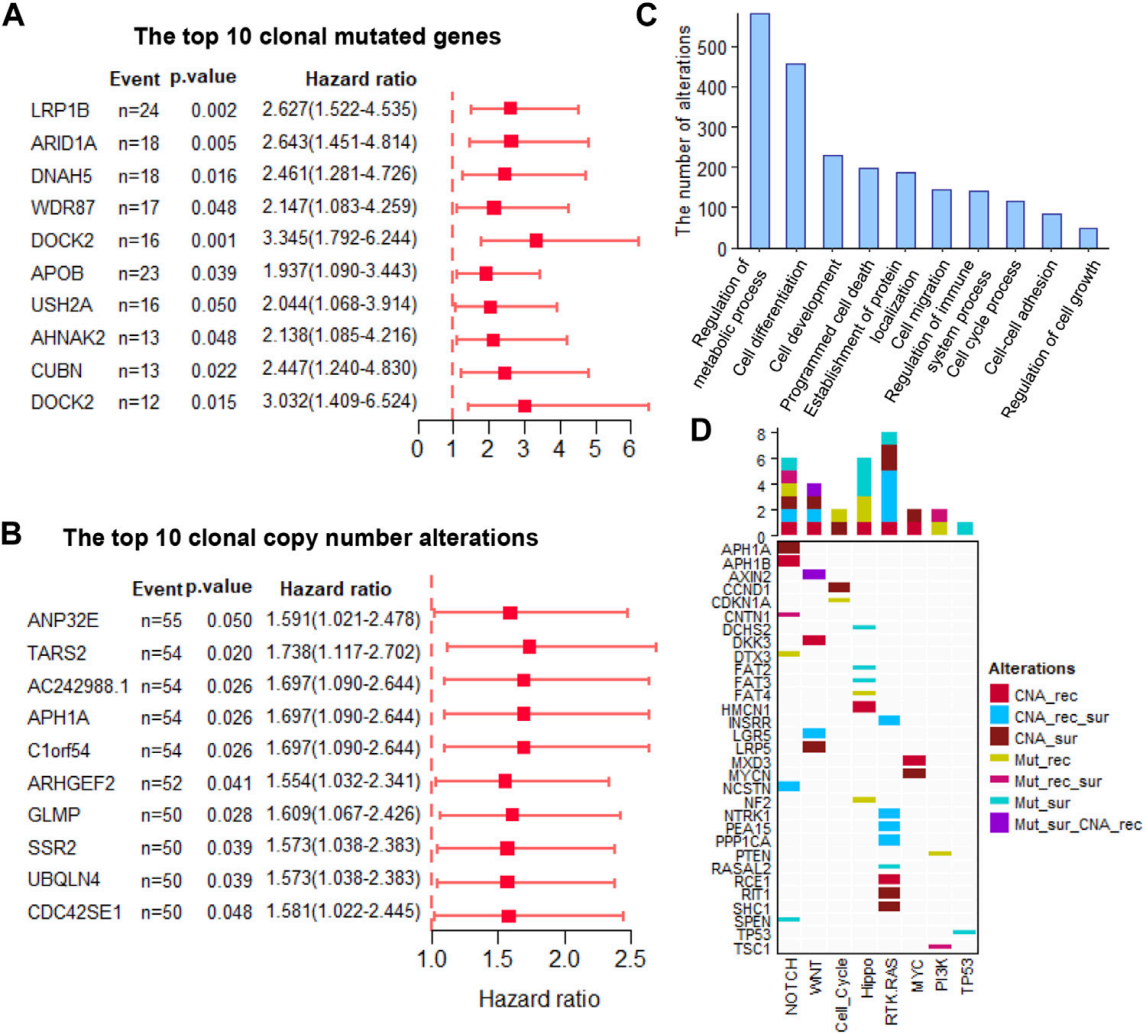
The prognosis-related clonal altered genes **(A)**. The prognosis potential of the top 10 clonal altered somatic mutations **(B)**. The prognosis potential of the top 10 clonal altered CNAs **(C)**. Gene Ontology analysis of clonal altered genes **(D)**. The clonal altered genes involved in oncogenic pathways. CNA_rec, the clonal altered CNAs that related to recurrence; CAN_sur, the clonal altered CNAs that related to survival; CAN_rec_sur, the clonal altered CNAs that both related to recurrence and survival; Mut_rec, the clonal altered somatic mutations that related to recurrence; Mut_sur, the clonal altered somatic mutations that related to survival; Mut_rec_sur, the clonal altered somatic mutations that both related to recurrence and survival; Mut_sur_CNA_rec, the clonal altered somatic mutations and CNAs that related to survival and recurrence, respectively.

The clonal altered genes come from the most recent common ancestor (MRCA) of the diverse tumor cell populations in each tumor and are the common markers in all the tumor cells. These markers overcome the influence of ITH and are stably present in all tumor cells, which not only provided promising therapeutic targets but also stable prognostic biomarkers. Here, we assessed the power of all the clonal altered genes (including non-silent somatic mutated genes and CNAs) in predicting a patient’s survival and recurrence. Results showed that 60 and 89 clonal non-silent somatic mutated genes were related to overall and recurrence-free survival, respectively (Figure 2A and Supplementary Tables S1, 2). In addition, 544 and 505 clonal altered CNAs were related to overall survival and recurrence-free survival, respectively (Figure 2B and Supplementary Tables S3, 4). Gene Ontology analysis showed these prognosis-related genes significantly enriched in many tumor-related biological processes, e.g., regulation of the metabolic process, cell differentiation, cell development, cell migration, cell cycle process, and regulation of cell growth (Figure 2C). Furthermore, we found these altered clonal genes were involved in regulating eight oncogenic pathways, among which NOTCH, WNT, Hippo, and RTK-RAS enriched the most clonal changes (Figure 2D). These results revealed that the clonal altered genes play an important role in tumor development and prognosis.

### The prognosis-related genes in RNA expression level

**FIGURE 3:**
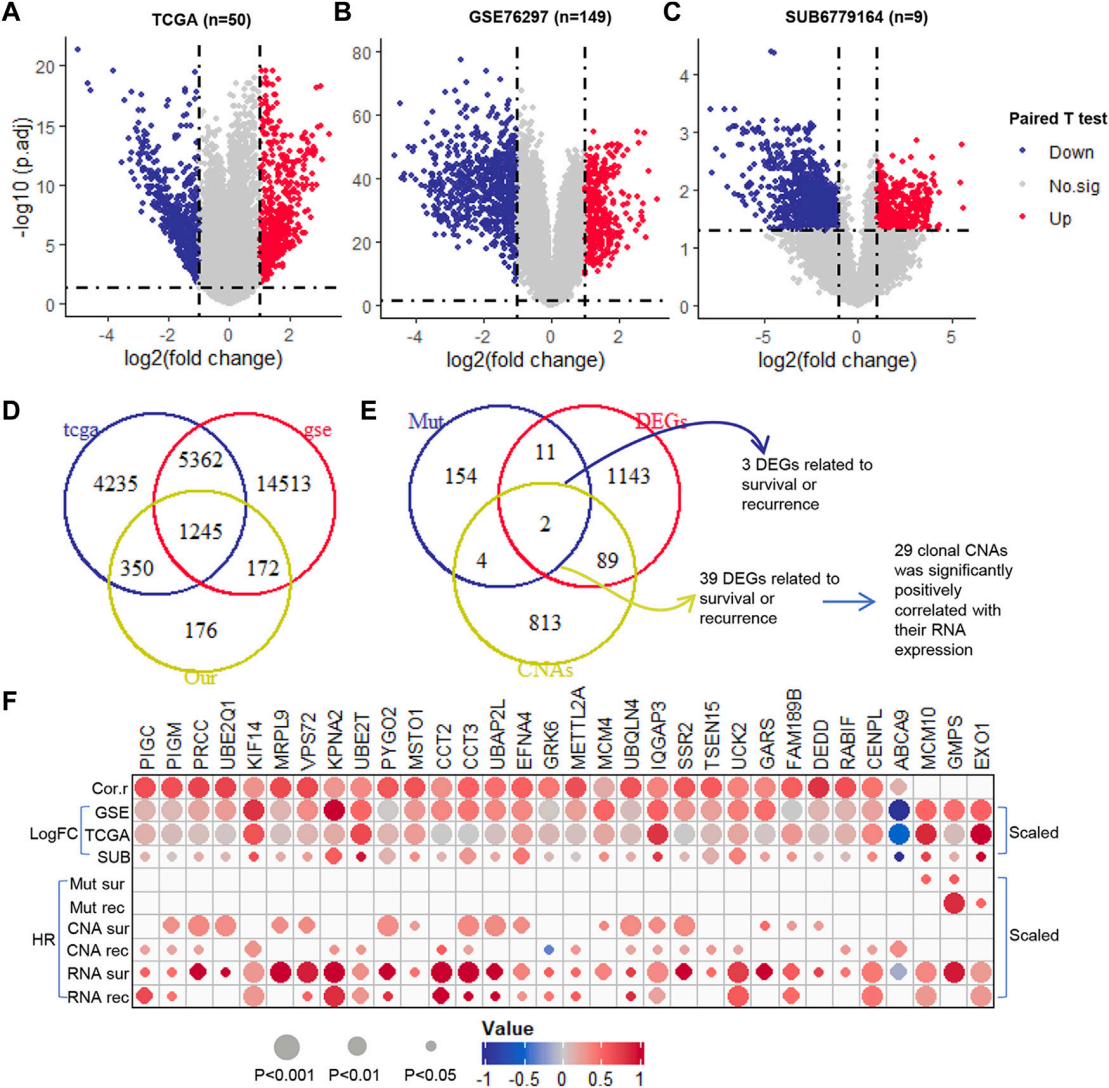
The prognosis potential of clonal altered genes in RNA level. **(A–C)**. The Volcano plot of DEGs in TCGA (50 paired tumor and normal samples), GSE76297 (149 paired tumor and normal samples), and SUB6779164 (9 paired tumor and normal samples) **(D)**. The Venn diagram of DEGs in TCGA, GSE76297, and SUB6779164 **(E)**. The Venn diagram of the selected DEGs, prognosis-related clonal somatic mutations, and CNAs **(F)**. The correlation between the genomic and transcriptomic levels of the candidate genes. Down, down-regulation; Up, up-regulation; No.sig, no significant difference; DEGs, differentially expressed genes; Cor.r, the correlation between the GISTIC score and RNA expression of the candidate clonal CNAs. LogFC, log2 (fold change) (which was scaled from −1 to 1); HR, hazard ratio (which was scaled from −1 to 1).

Using paired T-test, we identified the DEGs in paired normal and tumor samples at RNA expression level in TCGA (*n* = 50), GSE76297 (*n* = 149) and SUB6779164 (*n* = 9) (Figure 3A–C). All the genes with adjusted *p*-value <0.05 and their expression trend has been cross-validated in the three cohorts were selected for further analysis. Among that, 499 were up-regulated and 746 were down-regulated (Figure 3D). Furthermore, we evaluated the prognostic potential of these genes at transcription level. Results showed that 529 DEGs were significantly correlated with patients’ overall survival time (Supplementary Table S5), and 531 DEGs were significantly correlated with patients’ recurrence-free survival time (Supplementary Table S6). Notably, only 3 of them were clonal somatic mutations, and 29 of them showed a significantly positively correlated with their GISTIC score (Figure 3E). Finally, these 32 clonally altered genes both with prognosis potential in genomic and transcription levels were selected as candidate genes (Figure 3F).

### Functional enrichment analysis of the clonal expression genes

**FIGURE 4:**
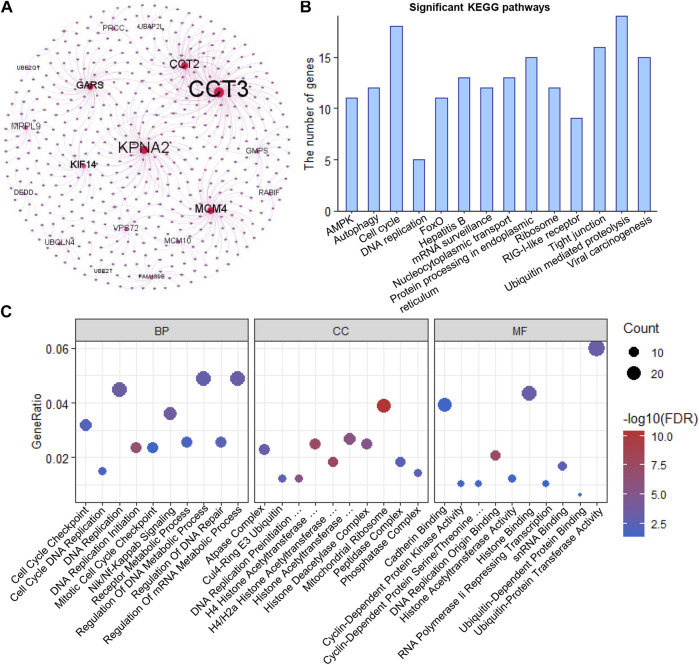
Functional enrichment analysis **(A)**. PPI network of the clonal alterations **(B)**. KEGG pathway analysis **(C)**. Gene Ontology analysis.

Using HINT, we identified 460 specific genes which showed direct protein-protein interaction (PPI) with these 32 clonal expression genes (including 1,138 PPI pairs) and constructed a PPI network (Figure 4A). Further analysis showed these genes participated in multiple cancer-related pathways regulation, such as AMPK, autophagy, cell cycle, DNA replication, FOXO, and Viral carcinogenesis signaling pathways (Figure 4B). Gene Ontology analysis showed these genes were significantly enriched in biological process (Cell Cycle Checkpoint, Nik/Nf-Kappab signaling, DNA replication initiation, regulation of DNA repair and metabolic process), Cellular Component (atpase, histone acetyltransferase, and DNA replication preinitiation complex) and Molecular function (Histone binding, Cyclin-Dependent protein kinase activity, and cadherin binding) (Supplementary Table S7). All of that indicated that the clonal expression genes play critical roles in tumor development (Figure 4C).

### Clinicopathologic correlation analysis

**FIGURE 5:**
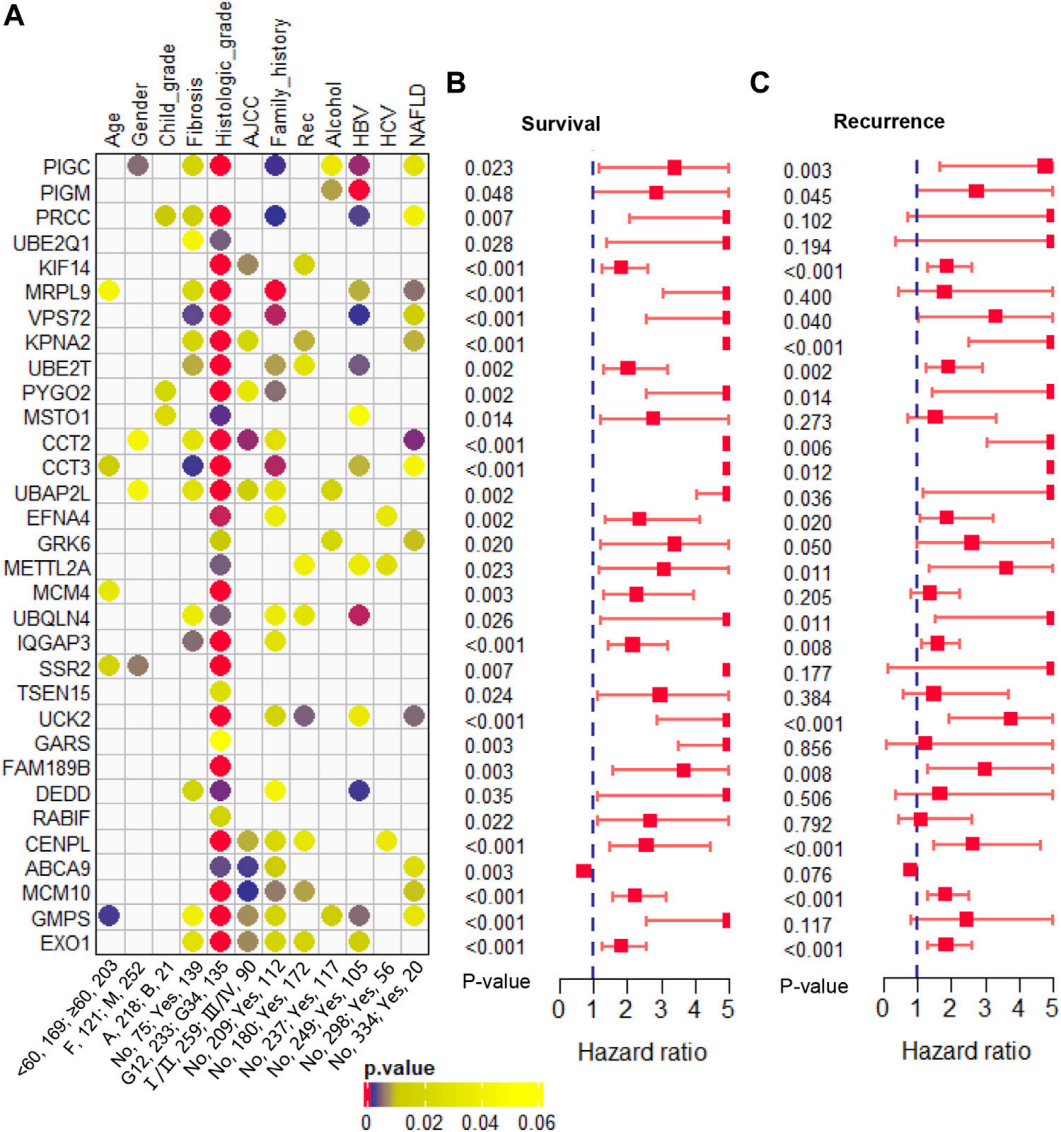
Clinical phenotype analysis **(A)**. The correlation of the candidate genes and clinical phenotypes **(B)**. The hazard ratio and *p*-value of the candidate genes in overall survival predicting **(C)**. The hazard ratio and *p*-value of the candidate genes in recurrence-free survival predicting.

Furthermore, we evaluated the correlation between these 32 genes and clinical phenotypes. Among that, three genes (*PRCC*, *PYGO2*, and *MSTO1*) were related to Child-pugh grade; 15 genes were related to liver fibrosis; 31 genes were related to histologic tumor grade; 10 genes were related to AJCC stage; 19 genes were related to family history of cancers; and 9 genes were related to tumor recurrence (Figure 5A). In addition, we analyzed the correlation between these clonal expression genes and risk factors for liver cancer. Results showed five genes (*PIGC*, *PIGM*, *UBAP2L*, *GRK6*, and *GMPS*) were related to alcohol; 14 genes were related to HBV infection; three genes were related to HCV infection; and 12 genes were related to NAFLD (Figure 5A). All of the clonal expression genes showed a significant correlation with overall survival time (Figure 5B). 21 of clonal expression genes showed a significant correlation with recurrence-free survival time (Figure 5C). These results revealed that these candidate genes were significantly correlated with clinical phenotypes.

### Multivariate cox regression model for predicting prognosis

**FIGURE 6:**
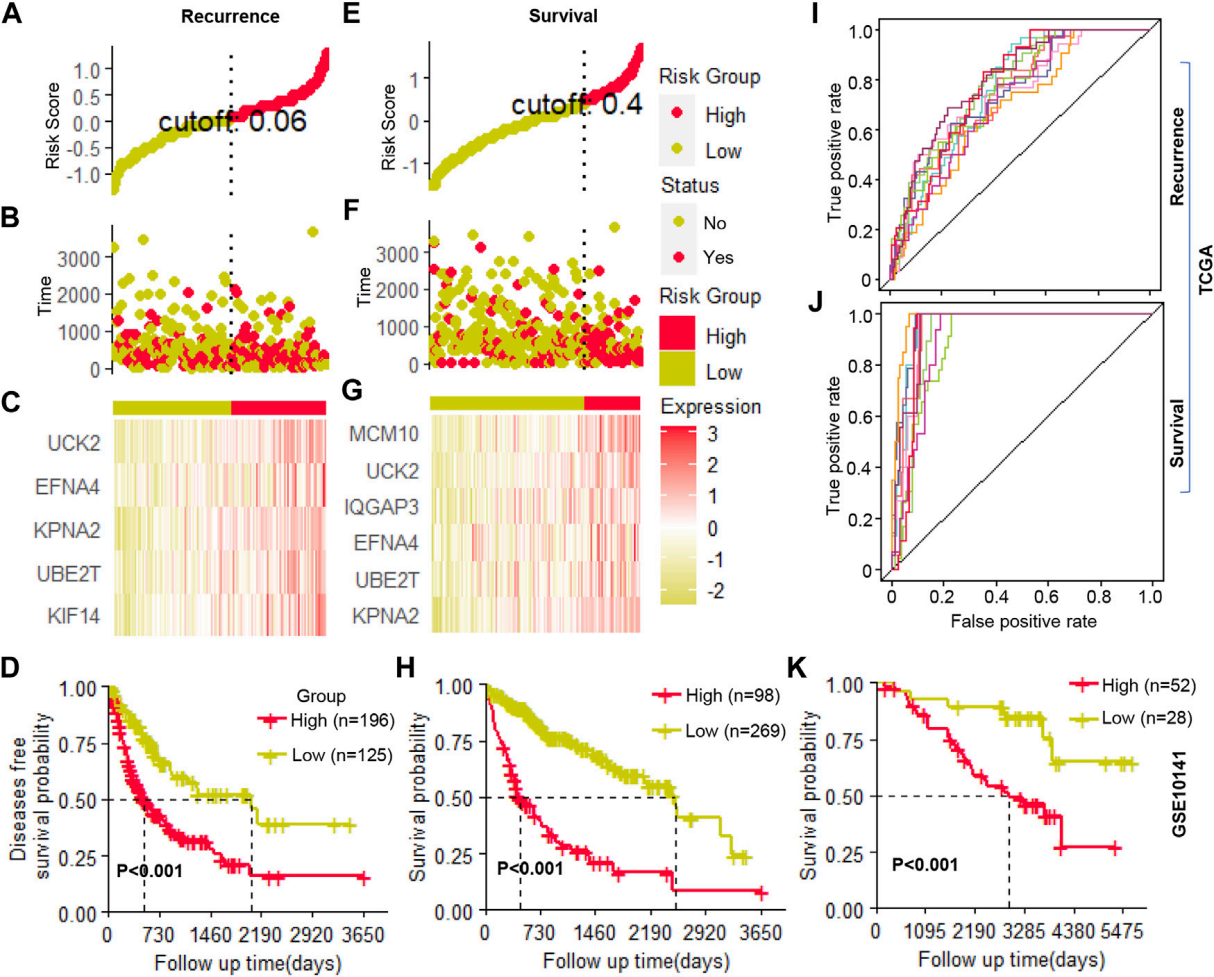
Cox regression model construction **(A–C)**. The cutoff value selection, patient stratification, and clonal gene expression in the recurrence model **(D)**. The Kaplan-Meier (KM) curve of patients’ recurrence-free survival **(E–G)**. The cutoff value selection, patient stratification, and clonal gene expression in the survival model **(H)**. The Kaplan-Meier (KM) curve of patients’ overall survival **(I,J)**. The ROC curve of ten random repetitions in recurrence and survival models **(K)**. The Kaplan-Meier (KM) curve of patients’ overall survival in an independent cohort.

The candidate genes that showed prognosis potential in overall and recurrence-free survival were used to construct a multivariate Cox regression model for survival and recurrence predicting, respectively. Using stepwise regression, five (*UCK2*, *EFNA4*, *KPAN2*, *UBE2T*, and *KIF14*) and six (*MCM10*, *UCK2*, *IQGAP3*, *EFNA4*, *UBE2T*, and *KPNA2*) clonal expression genes were finally selected for recurrence and survival model construction, respectively (Figure 6A–C and Figure 6E–G, respectively). In the recurrence model, the KM plot showed that the time to recurrence in 50% of patients was about one to 2 years in a high-risk group and five to 6 years in the low-risk group (Figure 6D). In the survival model, the time to death of 50% of patients was about one to 2 years in high-risk group and six to 7 years in the low-risk group (Figure 6H). The formula was described as follows:

H(t) = H0(t)exp (0.008*(KIF14*(t/365 + 1) −0.014*(UBE2T*(t/365 + 1) + 0.033*KPAN2 + 0.019*EFNA4 + 0.025*UCK2) (Recurrence module)

H(t) = H0(t)exp (0.034*(KPNA2+ln (t + e) −0.050 *(UBE2T + ln (t + e) + 0.027 * (EFNA4+ln (t + e) + 0.011*(IQGAP3+ln (t + e)+ 0.030 * (UCK2+ln (t + e) + 0.031 * (MCM10 + ln (t + e)) (Survival module)

Where H (0) corresponds to the baseline risk; where t corresponds to the time the event occurs; gene name corresponds to its expression value; e corresponds to natural constants; exp corresponds to the exponents of natural constants; ln corresponds to the natural logarithm. Then, using the selected clonal expression genes in the stepwise regression, we constructed a multivariate Cox regression model in randomly selected 70% samples of TCGA, and the remaining 30% samples were used to validate the accuracy of the models. In ten random repetitions, the survival and recurrence models showed high repeatability which significantly distinguished patients with high or low risks in each train and validation cohort (Figure 6I, J, Supplementary Table S8). In addition, we constructed a multivariate Cox regression model in an independent cohort by the clonal expression genes, which significantly identified high- and low-risk patients (Figure 6K). All of the results indicated that these candidate clonal expression genes with advantages in predicting patients’ outcomes.

## Discussion

Liver cancer exhibits high levels of ITH (Zhai et al., 2017; Ding et al., 2019; Xu et al., 2019), which not only brings challenges to tumor treatment (Bruix et al., 2015; Kelley, 2015; Niu et al., 2017; Xu et al., 2019) but also significant sampling bias in tumor research (Zhai et al., 2017; Ding et al., 2019). Genomic alterations are the main factor regulating gene expression. Therefore, the ITH at the transcription level cannot be ignored. Studies have demonstrated significant heterogeneity at the transcriptomic level (RNA-ITH) in multiple cancer types, which has been shown to confuse existing expression-based biomarkers (Gulati et al., 2014, 2015; Gyanchandani et al., 2016; Lee et al., 2018; Biswas et al., 2019). In the present study, we identified a set of clonally (i.e., present in all tumor cell populations) altered genes and also expressed significantly, which might be potential stable (i.e., overcoming spatial ITH) biomarkers in prognosis.

Clonal altered genes have important prognostic value, due to their founder roles in driving malignant transformation, and without spatial ITH. Firstly, we identified 867 clonal altered somatic mutations and 6,613 CNAs. Consistent with previous studies that the prevalence of clonal altered CNAs was significantly higher than subclonal altered CNAs in a patient, indicating most of the CNAs occurred early during tumor development (Navin et al., 2011; Wang et al., 2014; Duan et al., 2018). Previously well-documented HCC drivers (*TP53*, *CTNNB1*, *TTN*, *MUC16*, and *APOB*) were observed to present as clonal alterations, consistent with their essential role in tumor initiation. Then, we evaluated the prognosis potential of these clonally altered genes. Results showed that 258 clonal somatic mutations and 1,477 clonal CNAs were significantly related to patients’ prognosis. Gene Ontology analysis revealed that these clonally altered genes participated in important tumor-related biological processes, e.g., regulation of the metabolic process, cell differentiation, cell development, cell migration, cell cycle process, and cell growth. In addition, these genes are involved in the disorder of eight key oncogenic pathways (Sanchez-Vega et al., 2018). These findings highlight that clonally altered genes play an important role in tumor progression. Accurate identification of prognostic-related clonal altered genes may bring new insights for tumor-targeted therapy and prognostic evaluation.

A previous study revealed that the transcriptomic biomarkers that overcome tumor sampling bias and associate with survival independent of clinicopathological risk factors are often clonal in somatic mutations or CNAs (Biswas et al., 2019). Here, 32 clonal altered genes in genomic (29 clonal CNAs and 3 clonal somatic mutations) also significantly expressed in transcription level with strong prognosis potential were the candidate markers for better prognosis predicting. Functional enrichment analysis revealed these clonal expression genes play crucial roles in tumor development (e.g., cell cycle, DNA replication, and metabolic process). Consistently, *IQGAP3* was reported to promote cell proliferation through Ras/ERK signaling (Nojima et al., 2008) and predicts poor prognosis in multiple cancer types (Shi et al., 2017; Cao et al., 2019; Hua et al., 2021); *UBE2T* was reported as an oncogene, which is involved in cell-cycle modulation and related to poor prognosis (Ueki et al., 2009; Wang et al., 2016; Liu et al., 2017; Zhang et al., 2019); *SSR2* promote tumorigenesis and metastasis through modulating EMT (Hong et al., 2020). Further analysis revealed that these clonal expressed genes were significantly correlated with clinical phenotypes. Taken together, these findings highlight that these candidate genes may promote a poor prognosis through their important biological roles.

Furthermore, the multivariate Cox regression models constructed by clonal expression genes showed a significant power in prognosis prediction. In the survival model, the survival rate of patients who were identified as high-risk group dropped to 50% in about one to 2 years, while it took six to 7 years in a low-risk group. Similarly, in the recurrence model, the recurrence-free survival rate in the high-risk group drop to 50% about one to 2 years, and 5 to 6 years in a low-risk group. All of that suggested the high power of these clonal expression genes in prognosis prediction. Notably, the models constructed by the clonal expression genes kept high resolution in each random repetition, which may be related to clonal expression genes significantly reducing the misalignment of predictions caused by sampling bias. Hence, the models constructed based on clonal expression genes may have strong general applicability.

Notably, limited by the number of samples of multi-region sequencing in transcription level in liver cancer, whether these candidate genes overcome the effects of RNA-ITH needs further confirmation. Future work with a large cohort of multi-region sequencing at transcriptome level in liver cancer may further consolidate and extend the conclusions of our work. In addition, although the clonal altered genes provide information for the location of tumor cells, the acquired drug resistance caused by subclonal alterations also needs attention.

Taken together, our work provides a set of biomarkers that come from the tumor founder cell (i.e., clonal) in liver cancer for the first time and provides new insights for the design of prognostic markers that overcome the influence of spatial ITH.

## Data Availability

The original contributions presented in the study are included in the article/Supplementary Material, further inquiries can be directed to the corresponding author.
